# Interactive process mining of cancer treatment sequences with melanoma real-world data

**DOI:** 10.3389/fonc.2023.1043683

**Published:** 2023-03-21

**Authors:** Alexandre Wicky, Roberto Gatta, Sofiya Latifyan, Rita De Micheli, Camille Gerard, Sylvain Pradervand, Olivier Michielin, Michel A. Cuendet

**Affiliations:** ^1^ Precision Oncology Center, Department of Oncology, Lausanne University Hospital, Lausanne, Switzerland; ^2^ Dipartimento di Scienze Cliniche e Sperimentali dell'Università degli Studi di Brescia, Brescia, Italy; ^3^ Medical Oncology, Department of Oncology, Lausanne University Hospital, Lausanne, Switzerland; ^4^ Swiss Institute of Bioinformatics, University of Lausanne, Lausanne, Switzerland; ^5^ Department of Physiology and Medicine, Weill Cornell Medicine, New York, NY, United States

**Keywords:** precision oncology, process mining, real-world data, melanoma, immunotherapy, targeted treatment, treatment sequence

## Abstract

The growing availability of clinical real-world data (RWD) represents a formidable opportunity to complement evidence from randomized clinical trials and observe how oncological treatments perform in real-life conditions. In particular, RWD can provide insights on questions for which no clinical trials exist, such as comparing outcomes from different sequences of treatments. To this end, process mining is a particularly suitable methodology for analyzing different treatment paths and their associated outcomes. Here, we describe an implementation of process mining algorithms directly within our hospital information system with an interactive application that allows oncologists to compare sequences of treatments in terms of overall survival, progression-free survival and best overall response. As an application example, we first performed a RWD descriptive analysis of 303 patients with advanced melanoma and reproduced findings observed in two notorious clinical trials: CheckMate-067 and DREAMseq. Then, we explored the outcomes of an immune-checkpoint inhibitor rechallenge after a first progression on immunotherapy versus switching to a BRAF targeted treatment. By using interactive process-oriented RWD analysis, we observed that patients still derive long-term survival benefits from immune-checkpoint inhibitors rechallenge, which could have direct implications on treatment guidelines for patients able to carry on immune-checkpoint therapy, if confirmed by external RWD and randomized clinical trials. Overall, our results highlight how an interactive implementation of process mining can lead to clinically relevant insights from RWD with a framework that can be ported to other centers or networks of centers.

## 1 Introduction

Clinical evidence about treatment efficacy in oncology derives mostly from clinical trials, traditionally comparing one treatment to another in terms of overall survival (OS) or progression-free survival (PFS) probabilities. Nowadays, as a consequence of healthcare digitization (1), real-world evidence (RWE) is emerging as a complementary source of information to observe how treatments perform in real life conditions (2, 3). RWE is defined by the U.S. Food and Drug Administration (FDA) as the clinical evidence about treatment efficacy or usage obtained from various medical data sources called real-world data (RWD) that do not originate from traditional randomized clinical trials (RCTs) (4). In addition to providing useful information about treatment efficacy in the large share of patients that do not meet the strict trial selection criteria, the analysis of RWD can provide further information not available from clinical trials. In particular, it allows exploring the optimal sequence of treatments when no evidence of superiority between treatments exists.

During the last decade, the therapeutic arsenal against metastatic melanoma has dramatically evolved with the development of immune checkpoint inhibitors (ICI) and targeted treatments (TT) that have substantially improved patient prognosis. The ICI Ipilimumab (a human antibody targeting the cytotoxic T-lymphocyte-associated protein 4, CTLA4) was the first to demonstrate an OS benefit among patients with metastatic melanoma and was approved by the FDA in the beginning of 2011 (5–7). Few months later, a TT drug called Vemurafenib (a BRAF inhibitor, BRAFi) also demonstrated clinical benefits for melanoma patients having an actionable BRAF V600E/K mutation and it rapidly became standard of care for these patients, which represent about half of all metastatic melanomas (8–11). To note, BRAFi are now given in combination with MEK inhibitors (MEKi), which increase their efficacy and reduce their toxicity (12). In 2014, two ICIs targeting the programmed cell death 1 (PD1) protein, called Pembrolizumab and Nivolumab were also approved by the FDA (13–18). Efficacy of Ipilimumab, or Nivolumab, or the combination Ipilimumab+Nivolumab was evaluated with the phase III RCT Checkmate 067 that reported superior OS and PFS outcomes for patients receiving Nivolumab or the combination over Ipilimumab, with some patients reaching long-term complete responses (16), which represented a revolution compared to the dismal prognosis previously associated with chemotherapy.

Until recently there was no head-to-head comparison between TT and ICI, and we were missing a RCT that could assess the optimal sequence of treatments between these two options. This question was answered by the prospective phase III RCT called DREAMseq that assessed the optimal sequence of treatments between ICI and TT for patients with advanced melanoma harboring a V600 BRAF mutation (19). In this trial, treatments were switched upon progression, resulting in two arms: CTLA4+PD1 (Ipilimumab+Nivolumab) followed by BRAFi+MEKi (Dabrafenib+Trametinib) after progression, and the inverse sequence for second arm with BRAFi+MEKi followed by CTLA4+PD1. Preliminary results were recently published and showed a biphasic pattern with better OS and PFS responses in the initial 6 months for the arm starting with TT but significantly better long-term responses for the arm starting with ICI. Thus, the DREAMseq results favor ICI as a starting treatment, followed by TT if progression occurred. Interestingly, those results are in line with a previous analysis of RWD using the Flatiron framework and comparing the first lines ICI *vs* TT for advanced melanoma (20).

Despite the accumulation of evidence in metastatic melanoma with the trials described above, we are still lacking clear evidence from prospective RCTs on whether rechallenge of ICI after disease progression on a first ICI line could still be beneficial and provide longer survival than switching to another treatment kind. A few retrospective studies have reported a possible clinical benefit of the ICI rechallenge (21–23). Interestingly, one *post-hoc* analysis of the phase III KEYNOTE-006 trial (13) showed that from the 15 patients retreated with Pembrolizumab at progression after an initial response or stable disease with the same treatment, 8 of them had an objective response to rechallenge. Although this RCT was not specifically designed to evaluate ICI rechallenge, it may indicate that selected patients could still derive clinical benefits of it. Such questions on sequences of treatment lines are typically an area where RWD can provide the first insights that may then lead to the conception of RCTs.

The most common approach to explore the aforementioned questions exploits the standard Statistics toolbox (e.g. Kaplan-Meier curves, log-rank tests, Cox proportional hazard models, etc.) or the more advanced tools provided by Artificial Intelligence and Machine Learning. Here, in particular, many approaches arose in the last few years, such as decision trees, Bayesian networks or artificial neural networks and are growing in popularity thanks to the promising results they provide in coping with big and heterogeneous data. However, all these approaches are not typically able to capture the longitudinal complexity of the entire patients’ clinical pathways and often rely on many a-priori assumptions by the data analyst or the domain expert. Some paradigms able to mitigate such limitations can be seen in some time-series analysis approaches or in the case-based reasoning, but they are not radically oriented towards analyzing clinical pathways in the full complexity inherent to collected RWD. To bridge this gap, originating from Business Process Modeling (BPM), a relatively young discipline called Process Mining (PM) (24, 25) emerged and was then expressly adapted for clinical pathways and other healthcare-related processes. Process Mining for Healthcare (PM4HC) (26–28) is growing in popularity thanks to the central concept of the approach: Using real-world data as input, PM4HC can automatically represent the complexity of the true clinical processes without any a-priori knowledge. This paradigmatic shift from data mining to process mining allows investigators to make few assumptions and analyze processes in the entire space of possible clinical pathways.

In the last years, many reviews of PM4HC have been proposed, some general and some focused on different clinical application fields, such as in primary care or oncology (27, 29–31). However, while the potential of PM to support clinical research seems promising, it has so far been mostly limited to one-time retrospective analyses. To our knowledge, PM4HC has not yet been nested within the hospital IT infrastructure and made readily usable in the daily clinical practice and research activity. Moreover, it is still unclear how the different families of PM techniques could be implemented in such an integrated tool in order to reduce the gap between clinical research and clinical activity. Currently, in the literature, there is no evidence of a fruitful integration of such analysis tools into the existing hospital IT infrastructure and this application field remains unexplored in terms of pitfalls and documented benefits.

In the present paper, we describe an interactive web-based PM application to analyze the therapeutic journeys in custom melanoma patient cohorts from the Lausanne University Hospital (CHUV). Cohorts can be assembled in an interactive data curation and exploration environment that is directly integrated within the institutional clinical research data warehouse. The PM application automatically generates a tree representation of treatment lines called the Treatment Tree. It also displays treatment outcomes such as the best overall response (BOR), OS and PFS. Using drag-and-drop operations, users can generate complex patient sub-groups from different workflow nodes, and compare the corresponding aggregated OS and PFS. For demonstration, we focus on a cohort of advanced melanoma patients treated between 2011 and 2021 and present three different analyzes. First, we compare OS and PFS outcomes of the pivotal phase III RCT CheckMate 067 (16) with corresponding real-world data from our hospital. Second, we reproduce results of another prospective RCT investigating the relative benefits of different sequences of targeted and immune therapies (DREAMseq) (19). Finally, we show how our PM tool can be used to generate novel hypotheses by comparing melanoma patients that progressed after a first line of ICI and who were treated with either a targeted treatment (BRAF inhibitors) or with an ICI rechallenge. We thus demonstrate how our integration of process-oriented analysis within the hospital information system can unlock the potential of PM for clinical researchers.

## 2 Methods

### 2.1 Data curation environment

We developed a suite of applications called SQL-Tools, offering a user-friendly user interface for curating high-quality oncological patient data based on raw electronic patient records. Each tool is a Java-based web front end using advanced CSS and JavaScript features of the Chrome web browser and is deployed on a virtual machine within the hospital intranet. The back-end is hosted directly in the institutional Oracle clinical research data warehouse and communicates with the front-end *via* SQL queries (hence the name *SQL-Tools*). User access was secured with institutional OpenId authentication and the use of clinical data was approved by the local ethics committee (CER-VD) for patients who did not oppose data usage for research purposes.

**Figure 1:**
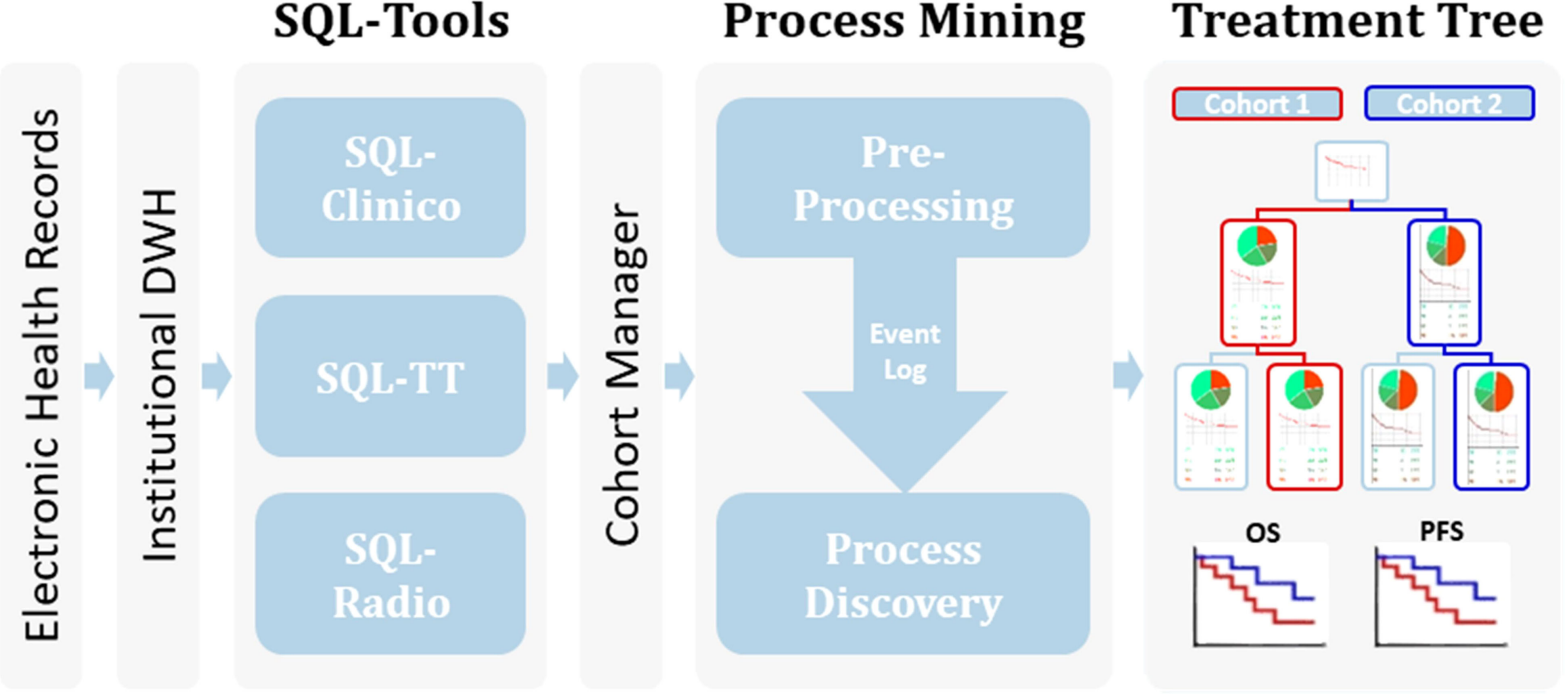
Workflow of the process mining analysis. Relevant clinical information from the EHR available within an Institutional Data Warehouse (DWH) system was curated in our SQL-Tools that include single-valued clinical data inherent to the patient or the tumor (SQL-Clinico), treatment data (SQL-TT) and radiological or clinical assessments of treatment responses (SQL-Radio). After cohort selection through the Cohort Manager, the associated clinical data was pre-processed to generate an Event Log, which was passed to a process discovery step to produce the interactive Treatment Tree. By using the Treatment Tree interface oncologists can select groups of patients that underwent a specific treatment path and compare their OS and PFS with other groups by simply drag-and-dropping the treatment boxes.

The environment includes the following modules (as described in **Figure 1**): SQL-Clinico allows saving single-value patient characteristics such as diagnosis, diagnosis date, recurrence date, brain metastasis and its date, TNM, stage and molecular alterations such as somatic mutations. SQL-TT is a smart interface for consolidation of discrete treatment lines with start and stop dates, and categorization into subtypes: CTLA4 (for the ICI Ipilimumab targeting CTLA-4), PD1 (for the ICI Nivolumab or Pembrolizumab targeting PD-1), BRAFi (for the targeted treatments Dabrafenib, Vemurafenib and Encorafenib which are BRAF inhibitors), MEKi (for the targeted treatments Trametinib, Cobimetinib, Binimetinib which are MEK inhibitors) and chemo (e.g. Dacarbazine and Temozolomide) as exemplified in **Table 1**. The treatment data also includes whether the drug was given in the adjuvant setting as for Line 1 in **Table 1**. Treatment maintenance as with the Nivolumab following Ipilimumab+Nivolumab induction in **Table 1** Line 2 was considered as a modification of Line 2. Similarly, for Line 3, after a switch of targeted treatments due to toxicity for example, the newly given treatments were not considered as a new line of treatment but as a modification of the same line because their treatment types remained identical. To note, the pre-processing step that generates an Event Log from the treatment data will merge the Line modifications and retain the first start date and latest stop date.

**Table 1:** Oncological treatment format in SQL-TT.

Patient ID	Line	Modification	Treatment	Type	Start	Stop	Setting
1234	1	1	Pembrolizumab	PD1	01.07.2016	15.12.2016	adjuvant
1234	2	1	Ipilimumab+Nivolumab	CTLA4+PD1	01.01.2017	01.03.2017	
1234	2	2	Nivolumab	PD1	15.03.2017	31.12.2017	
1234	3	1	Dabrafenib+Trametinib	BRAFi+TT_MEKi	15.01.2018	15.02.2018	
1234	3	2	Vemurafenib+Cobimetinib	BRAFi+TT_MEKi	01.03.2018	31.12.2018	

In addition, surgical procedures involving the tumor and radiotherapeutic treatments were also included but were not explored in detail in this paper. SQL-Radio was used for assigning disease progression or response scores similar to the RECIST criteria (Response Evaluation Criteria in Solid Tumours) (32). The attributes were: PD for progression disease, SD for stable disease, PR for partial response, CR for complete response and were assessed using the radiological examinations (CT, MRI and PET scans) and clinical evaluations that occur during treatments recorded in SQL-TT. RE labels were also used to define recurrence of disease and NR for no recurrence of disease specifically for adjuvant treatments. Note that the exact RECIST criteria could not be applied here, as the lesion measurements were not always available. To facilitate annotation, radiological exams (CT, PET-CT and MRI) together with clinical radiology reports were imported from the EHR, and oncologists assessed the treatment responses with the help of a natural language processing algorithm that detects possible progression events^1^. In rare cases, when no radiological examinations were documented, a clinical progression could still be entered manually when the information was available in the EHR. Finally, the **CohortManager** application is a module for dispatching cohorts to SQL-Clinico, SQL-radio, SQL-TT and launching the PM application to display the Treatment Tree.

### 2.2 The interactive process mining application

**Figure 2:**
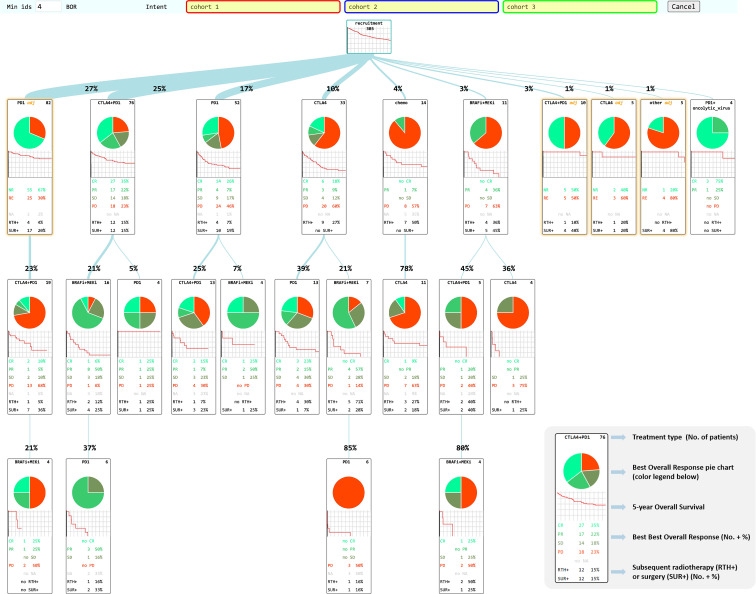
CHUV-RWD Melanoma Treatment Tree. Each box represents a new treatment line and the thickness of the branch depends on the percentage of patients from the parent box undergoing the next drug treatment. The legend in the bottom right corner details the information contained in each box of the tree. Adjuvant treatments are outlined in yellow. The red, blue, and green boxes at the top allow drag-and-drop custom cohort selection to generate OS and PFS curves as shown in the following sections. Each Sub-groups of patients detailed in each box can be accessed through a right-click and examined in the SQL-Tools or added to/subtracted from a custom cohort.

Our PM application used features from the pMineR software library in R (33), which was specifically designed to support PM investigations in healthcare. It supports researchers in Process Discovery exploiting first order Markov models and the CareFlow Miner algorithm (34). It also provides tools for Conformance Checking with a native and easy-to-use language for Computer Interpretable Clinical Guidelines implementations (35). The Process Discovery algorithm chosen for our integration was CareFlow Miner (CFM), a framework specifically designed to be intuitive for healthcare providers, who can be unfamiliar with other formalisms such as the Petri Net, for example. It uses an Event Log (a table containing in each row a clinical event, associated with the related patient ID and a time stamp) as input and produces a tree with a common starting point, called *root* or *recruitment* in **Figure 2**. The root node branches to all the possible first treatments as they appear in the input data, assigning in each node the information about the patients passing through it. For each of these first-line nodes, a new branch was built for each second-line treatment found in the associated sub-cohort. The approach was recursively applied to grow a full tree of all treatments performed on all patients in the cohort.

While existing CareFlow Miner implementations require programming skills for data pre-processing and for workflow analysis, the PM application presented here is a substantial improvement in these two areas. First, the pre-processing step to generate a clean Event Log from the initial dataset has been fully automated; in consequence, the Treatment Tree can be obtained with a single click after that the cohort has been selected in SQL-CohortManager. This was made possible by the tight integration within our data curation environment. Note however, that this is not a limitation of our tool, which could straightforwardly be adapted to accept any duly formatted Event Log as input.

Second, the Treatment Tree is fully interactive. For example, in order to control the complexity of the tree, the user can interactively select a patient number threshold under which weekly-populated nodes are omitted from the representation. Beyond the type of treatment administered, each node displays complex additional information, such as associated surgical interventions, radiotherapy and response to treatments in the form of a BOR pie chart and a thumbnail five-year OS Kaplan-Meier plot. The central feature that allows oncologists to experiment freely with the data is the front-end drag and drop option to create custom cohorts combining patients present in different treatment boxes. Note that patients can also be subtracted from a sub-cohort with a right click option on the corresponding box. Sub-cohorts can also be sent back to the SQL-Tools for data inspection or further curation. Finally, OS and PFS Kaplan Meier curves from the custom cohorts are automatically generated using an embedded R package (survival). The start of the survival analysis is automatically set to the start of the treatment from which the patients were selected. OS and PFS curves are displayed below the Treatment Tree with other metrics such as the age at treatment start, sex ratio and BOR for every sub-cohorts. The generated data can also be exported in excel format in order to save them. These results can directly be used for comparison to RCTs or for generating new research hypotheses, as we show in the Results section.

## 3 Results

### 3.1 Structure and usage of the application

Electronic Health Records (EHR) collected within an Institutional Data Warehouse system were processed using our suite of web-based curation applications called SQL-Tools, as described in the Methods section (**Figure 1**). The resulting consolidated dataset with diagnosis, treatments and radiological responses was quality-controlled by trained oncologists. The melanoma cohort was selected, and our PM application automatically generated the Treatment Tree shown in **Figure 2**. Patients receiving the same treatment or sequence of treatment were grouped in workflow nodes represented by boxes with information about the corresponding sub-cohort, including a Best-Overall Response (BOR) pie chart and a five-year OS curve since treatment initiation. The Treatment Tree is interactive, and oncologists with no specific technical training can test hypotheses by selecting custom sub-cohorts of patients that went through different sequences of treatments using a drag and drop option and compare their OS and PFS curves that are automatically generated with the relevant statistics. As the PM application is interconnected with our SQL-Tools, the user can easily go back and compare the clinical characteristics of each newly selected cohort with a one-click access to the SQL-Clinico, SQL-TT, or SQL-Radio applications.

### 3.2 Global structure of the treatment tree for CHUV advanced melanoma patients

A total of 303 non-uveal melanoma patients met the following eligibility criteria: having received ICI or TT at the CHUV; having sufficient clinical data documented; no loss of follow-up and no second cancer diagnosis requiring systemic treatments. For these 303 patients, 608 treatment lines were categorized into the main categories: CTLA4, PD1, BRAFi, MEKi and chemo. We performed process-oriented analysis of this cohort and created an interactive Treatment Tree starting from the first oncological treatment through the many possible consecutive therapy lines, and showing at each step, how patients subsequently evolved in terms of BOR and OS (**Figure 2**). In order to display the tree in a compact form, a threshold of four patients minimum per treatment box has been applied. Boxes corresponding to treatments in the adjuvant setting are highlighted in yellow. Although BOR pie charts are not formally compatible with adjuvant treatments, we have decided to keep the pie charts, but with only two colors indicating in red, the percent of patients that had a recurrence and in green, the ones that did not.

The most prevalent non-adjuvant oncological treatments were PD1 (174 lines of treatment), followed by CTLA4+PD1 (150 lines), BRAFi+MEKi (92 lines), CTLA4 (63 lines) and chemo (23). The other treatments were mostly combinations of PD1 with an anti-LAG3 antibody or the Talimogene Laherparepvec oncolytic virus. One of the first observation is that this Treatment Tree reflects the global survival improvement obtained with the use of ICI compared to historical treatments such as dacarbazine (chemo). As another example, we can see that patients offered a CTLA4+PD1 combination after an initial adjuvant PD1 treatment appear to have a much decreased BOR and OS (first box on second row) compared to patients who had a CTLA4+PD1 first line (second box on the first row). This result seems counterintuitive, but we have to keep in mind that the patients who had CTLA4+PD1 after adjuvant PD1 happen to be all in the subset of patients (23%) that progressed after the adjuvant line. Overall, patients undergoing adjuvant PD1 therapy (first box on the first row) have a better overall survival than any other major first line treatment type, as expected. While this simple example illustrates the types of biases that can appear upon a precipitous interpretation of a Treatment Tree, mindful oncologists can use the tree in many different ways to examine real-world data of their institution.

### 3.3 CHUV-RWD *vs* Checkmate 067

**Figure 3:**
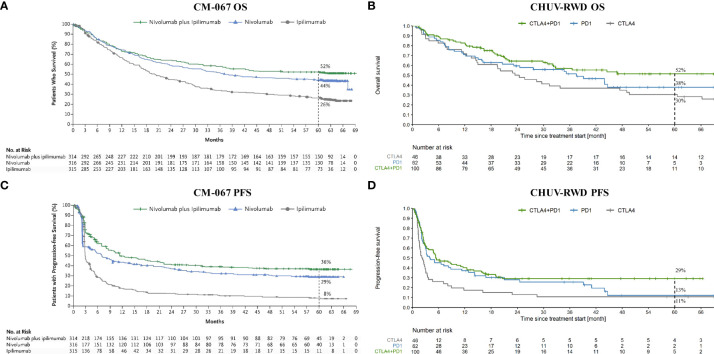
OS and PFS from Checkmate 067 (CM-067) and CHUV-RWD melanoma patients. Left panels **(A)** and **(C)** show the OS and PFS respectively from CM-067 and right panels **(B)** and **(D)**, OS and PFS for the CHUV-RWD. The left panels from CM-067 have been reproduced with permission from Larkin et al., 2019, NEJM (16). Dashed lines at 60 month indicate the percent of patients left in each group.

As a first application, we compared the OS and PFS of melanoma patients treated at CHUV with CTLA4, PD1, and the combination CTLA4+PD1, with the published results of the pivotal RCT Checkmate 067 (CM-067) that led to the approval of these treatments (Ipilimumab for CTLA4 and Nivolumab for PD1). As shown on **Figure 3**, we observe similar five-year OS and PFS curves for every treatment between CM-067 and the CHUV patients with the exception of the five-year PFS for PD1 (very few patients were still at risk at five years). Five-year OS rates were 26% *vs* 30% for CTLA4 (CM-067 and CHUV-RWD respectively), 44% *vs* 38% for PD1, and 52% *vs* 52% for CTLA4+PD1 patients (**Supplementary Table 1**). Five-year PFS rates were 8% *vs* 11% for CTLA4, 29% *vs*. 13% for PD1, and 36% *vs* 29% for CTLA4+PD1 patients. In particular, there was no statistically significant five-year survival difference between the CTLA4+PD1 and PD1 groups (HR: 0.72, 95% CI: 0.45 to 1.13; log-rank p-value: 0.15) but a similar trend was observed in both CHUV and CM-067, with a slightly superior efficacy of the ICI combination which returned the only statistically significant difference while comparing it with the CTLA4 group (HR: 0.55, 95% CI: 0.35 to 0.86; log-rank p-value: <0.01). In comparison, the hazard ratio for death in CM-067 was 0.52 (95% CI: 0.42 to 0.64; p-value<0.01) for the ICI combination versus CTLA4, 0.63 (95% CI: 0.52 to 0.76; p-value<0.01) for PD1 versus CTLA4, and 0.83 (95% CI: 0.67 to 1.03, p-value not published) for the ICI combination versus PD1. Interestingly, CHUV patients appear to have a five-year survival rate similar to the CM-067 trial for every treatment arm despite having different clinical characteristics. In particular, 31% of CHUV patients had brain metastases before treatment initiation, whereas it was an exclusion criterion for CM-67. In line with CM-067, the objective response rates (percentage of patients having partial and complete responses) were 53% for the combination of ICI, 42% for PD1 and 22% for CTLA4 (it was 58%, 45% and 19% respectively for CM-067). Those results are also in agreement with the latest published results of CM-067 (36).

### 3.4 CHUV-RWD *vs* DREAMseq

In a second step, we sought to replicate the DREAMseq trial using RWD from CHUV. As a reminder, this RCT tested the optimal sequence of TT and ICI treatments for patients with advanced melanoma harboring a V600 BRAF mutation. Treatments were switched upon progression, resulting in 2 arms: CTLA4+PD1→BRAFi+MEKi and the inverse sequence BRAFi+MEKi→CTLA4+PD1. However, in order to retrieve enough patients for our analysis, few adaptations were made. First, we had to adapt the ICI treatment to be any PD1-based treatment (PD1+/-CTLA4 instead of CTLA4+PD1 in DREAMseq). Second, for TT, we selected all BRAFi-based treatments (BRAFi+/-MEKi instead of the combination BRAFi+MEKi for DREAMseq). In addition, given the low impact on survival and progression-free survival of chemo regimens and oncolytic viruses compared to ICI and targeted treatments, those treatments were neglected in the sequence of treatments.

**Figure 4:**
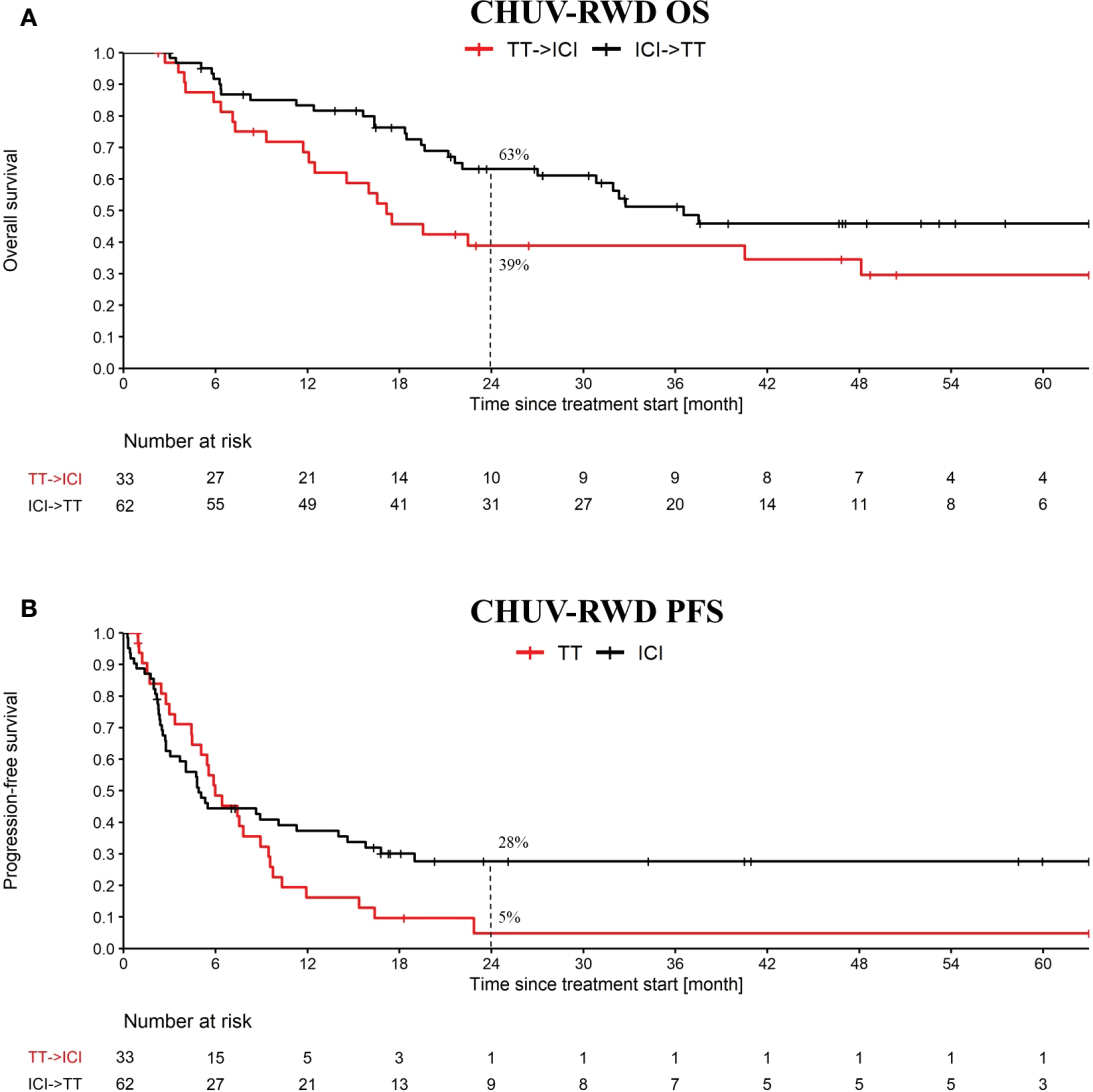
OS and PFS from the treatment sequence: targeted treatment (TT) followed by ICI and the converse. OS **(A)** and PFS **(B)** analysis starts at the first cure of the first treatment. The PFS analysis is only evaluating the first treatment. TT refers to targeted therapy (i.e. BRAFi+/-MEKi) and ICI refers to PD1+/-CTLA4. Dashed lines at 24 month indicate the percent of patients left in each group.

Our PM application is particularly suited to this use-case, since it implies sequences of treatments that can be immediately identified in the Treatment Tree. By drag-and-dropping the corresponding second-line nodes, we obtained the OS and PFS curves shown in **Figure 4**. Globally, we observe slightly worse OS and PFS curves at two years at CHUV for both arms compared to the one reported in DREAMseq, which can be explained by patient selection criteria, where DREAMseq included only patients with Eastern Cooperative Oncology Group (ECOG) performance status of 0 or 1 and excluded patients with active brain metastases for example. In addition, we included PD1 monotherapy and BRAFi monotherapy at the CHUV, which both could have a slightly lower efficacy than the corresponding combinations. Nonetheless, we observed a similar pattern between CHUV-RWD and DREAMseq with a 24% increase of overall survival at two years for the arm ICI→TT (63% patients alive, 95% CI 51 to 76) compared to the arm TT→ICI (39% patients alive, 95% CI 22 to 56) (**Supplementary Table 2**). This difference was similar to the one reported from DREAMseq with a 20% difference in favor of the ICI→TT arm (72% patients alive, 95% CI 63 to 79) *vs* TT→ICI arm (52% patients alive, 95% CI 42 to 60). Similar findings were observed for the PFS assessing the first line treatment and returning the same PFS difference of 23% at two years for DREAMseq and CHUV-RWD in favor the ICI→TT (42%, 95% CI 31 to 52 for DREAMseq and 28%, 95% CI 16 to 39 for CHUV-RWD).

### 3.5 ICI rechallenge

After showing that our PM analysis application can be used to reproduce the CM-067 and DREAMseq clinical trials with CHUV-RWD, we present one use case where the PM application is used to generate hypotheses about an important clinical question that emerges when considering the various treatment sequences shown on **Figure 2**. Namely, we investigated the question of the optimal second line treatment in metastatic melanoma patients bearing an actionable BRAF V600 mutation and progressing after a first line of immunotherapy. The standard guidelines suggest switching to a BRAFi+MEKi treatment but some oncologists persist with ICI and attempt to rechallenge the patients with a different line of immunotherapy.

**Figure 5:**
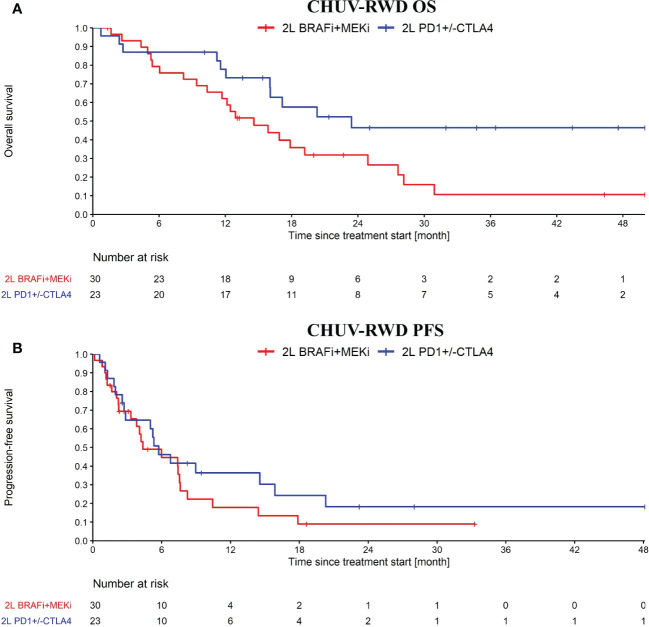
OS **(A)** and PFS **(B)** from ICI rechallenge *vs* switching to BRAFi+/-MEKi upon progression after a first line of immunotherapy. In blue, the ICI rechallenge group and in red the BRAF v600 mutant melanoma patients that switched to BRAFi+/-MEKi. Analysis started at 2nd line treatment initiation. Nivolumab maintenance was not considered as an ICI rechallenge. Patients receiving CTLA4 only, BRAFi+/-MEKi, PD1+Lenvatinib or chemo as first line treatment were excluded from this analysis.

After generating a cohort of melanoma patients in our SQL-Tools, we launched our PM application and drag-and-dropped the appropriate boxes to generate the OS and PFS plots shown in **Figure 5**. Interestingly, we observed a potential overall OS benefit of rechallenging with ICI (PD1+/-CTLA4) after an initial progression on ICI compared to switching to BRAFi+/-MEKi (HR: 0.46, 95% CI: 0.22 to 0.94; log-rank p-value: 0.03). Importantly, the majority of the ICI rechallenge consisted in PD1 followed by CTLA4+PD1 (65% of the patients). However, no significant difference was observed with the PFS (HR: 0.71, 95% CI: 0.38 to 1.35; log-rank p-value: 0.3). Note that to bolster our rechallenge cohort, we included 15 BRAF wild-type patients. Since these patients tend to have a slightly worse response to ICI compared to BRAF V600 patients (16), the possible bias introduced by including these patients would tend to underestimate the OS difference. While this result on ICI rechallenge is not strong enough per se to support clinical-grade conclusions and needs to be further validated, it illustrates how our PM application enables oncologists to quickly explore RWD and generate hypotheses for further investigations.

## 4 Discussion and conclusion

To exploit RWD in precision oncology, where patients undergo diverse and complex therapeutic journeys, powerful analytic tools are required. Over the last few years, PM has emerged as a method particularly relevant for addressing this need. However, deploying PM analysis for a particular research project required so far solid programming skills, even with packages such as the pMineR implementation in R. In the present work, we take PM to the next level by integrating the approach in an interactive dashboard that enables oncologists to explore RWD without any extra technical skills. In minutes, they can generate OS and PFS curves for arbitrary sub-cohorts selected based on their paths through the Treatment Tree.

In recent years, there has been a growing interest in dashboards for healthcare as demonstrated by the rise of publications including the keyword “dashboard” in PubMed, with 175 publications in 2018, 205 in 2019, 365 in 2020 and 518 in 2021. In 2022, 1254 articles on the theme were reviewed, outlining possible challenges and opportunities for the future (37). In a 2020 paper for example, dashboards were shown to be effective in providing interactive visualizations to support clinical or managerial purposes in the hospital (38). In three recent papers published in 2021 (39) and 2022 (40, 41), different design patterns and frameworks for dashboards were proposed but none included PM approaches for oncology. The only project found that included interactive dashboards with PM algorithms was published in 2019 and was intended to analyze processes in surgical rooms (42). Even though we found projects using PM approaches to explore oncological data, like the study of Kurniati et al. (43), which analyzed transitions between different hospital units during hospitalization of cancer patients, PM was not integrated as an interactive tool in the hospital system. Thus, our project is the first attempt to our knowledge to use PM in an interactive dashboard specifically for oncology.

To demonstrate the potential of our interactive PM application, we showed results for three use cases: we first established the relevance of our CHUV advanced melanoma dataset by defining sub-cohorts based on the first (non-adjuvant) line of ICI treatments, mimicking the pivotal Phase III clinical trial CM-067. Even though we had much fewer patients and with more advanced disease (CM-067 selected only patients with ECOG ≤ 1 and without active brain metastases), our results were qualitatively similar to CM-067. Interestingly, like in CM-067, we observed a trend for better survival probabilities with CTLA4+PD1 compared to PD1 single agent, even if the difference was not statistically significant. It is interesting to find this result in RWD since in routine practice the CTLA4+PD1 combination is generally preferentially given to patients with more advanced disease like with brain metastases and worse prognosis, which would tend to diminish its apparent efficacy. Thus, our results both validate the approach and provide an additional piece of evidence of the anti-CTLA4 added value, which should be confirmed with further RWD studies applying the same methodology to extended cohorts.

Secondly, we demonstrated the usefulness of our interactive PM application for analyses comparing sub-cohorts defined according to treatment sequences, which can be easily selected by collecting the corresponding paths in the Treatment Tree. We compared the outcomes of sequences ICI → TT *vs*. TT → ICI, which was the object of the recent Phase III clinical trial DREAMseq. The CHUV-RWD showed overall decreased PFS and OS compared to DREAMseq, but the gap between treatment sequences was very similar to that observed in DREAMseq. Hence, by using our tool we were able to confirm within minutes that the CHUV-RWD is consistent with the DREAMseq RCT, despite few differences in the selection criteria.

Third, we placed ourselves in a scenario where, instead of repeating existing clinical trials, our interactive PM application was used to mine RWD and generate new clinical hypotheses. We compared the OS and PFS of patients rechallenged with ICI to the standard practice that consists of switching for a targeted treatment in case of progression after first line ICI therapy. We observed interesting results with a trend towards more favorable survival benefit in the ICI rechallenge group. While transforming this trend into hard actionable evidence would admittedly require additional RWD from external datasets and/or proof in an RCT, this example shows the value of our PM application to guide future research or generate preliminary results for funding proposals.

Beyond the three applications reported here, many others come to mind. For example, with our PM application we could easily investigate whether a difference between PFS and OS can be explained by subsequent treatments received by some patients. Moreover, we could also detect whether radiotherapies or surgical treatments can rescue some patients that progressed in specific lesions that could be treated by these modalities. Overall, the ability for CHUV oncologists to harness PM to monitor their past patients and generate interesting research hypotheses has become very valuable. As a complementary benefit, the tight integration of PM with our clinical data warehouse and data curation tools enables data managers to use the PM application as a quality assurance tool. Indeed, any suspicious path in the Treatment Tree can be quickly investigated by returning to the source data with a single click, fixing any annotation mistake, and iteratively re-generating an updated Treatment Tree. The next step is to evaluate our tools with oncologists and implement the appropriate modifications according to their feedback in order to maximize the tool’s usability and acceptability in practice.

As it is the case for RWD in general, evidence from our PM application should be extrapolated carefully, as important biases may be inherent to the data and need to be controlled for. However, if such biases can represent a disadvantage compared to RCTs, exploiting diverse RWD is also an opportunity as some patient subgroups may be underrepresented in RCTs, whose inclusion criteria often do not represent the real-world patient diversity. A more practical limitation of our PM application is that it relies on appropriately curated data to build a clean Event Log and generate a meaningful Treatment Tree, such as the data provided by our SQL-Tools. This implies that the PM application will not work out of the box at another institution with different semantics and data formats, without adapting the data pre-processing stage. Nevertheless, with only few adaptations, the tool could be implemented within large data-sharing networks such as the upcoming Swiss Precision Oncology network led by CHUV (44, 45). When connected to such large-scale RWD sources, our PM application will see the breadth of its possibilities and the strength of the evidence generated skyrocket. As a next step, we will implement the possibility to select patients based not only on treatment sequence, but also on other clinical variables, such as demographics, laboratory values, genomics, and more. This additional capability will turn our PM application into an interactive discovery machine geared to finding digital biomarkers of response for specific treatment sequences, thus, serving directly one of the overarching goals of precision oncology.

## Data availability statement

The original contributions presented in the study are included in the article/**Supplementary Material**. Further inquiries can be directed to the corresponding authors.

## Ethics statement

This study was approved by the local ethics committee (CER-VD, protocol 2019-00448) for patients who provided written informed consent for or did not oppose retrospective usage of health-related personal data for research purposes. Written informed consent from the participants was not required to participate in this study in accordance with the national legislation and the institutional requirements.

## Author contributions

Clinical data collection: AW, SL, RDM, CG, SP. Methodological developments: AW, MC, RG. Data analysis: AW, MC, OM. Manuscript writing: AW, MC, RG, OM. All authors contributed to the article and approved the submitted version.

## Footnotes

1 The natural language processing algorithm applied to radiology report conclusions to detect possible progression was trained using a training set of 1000 previously annotated conclusions. A machine-learning model for automatic binary classification was adapted from the TensorFlow library https://www.tensorflow.org/text/tutorials/text_classification_rnn. The accuracy of the model evaluated on a validation set of 300 samples reached 92%. The accuracy on an external test set including 4975 samples was 86%.
