# Supplementary material for: Interactive process mining of cancer treatment sequences with melanoma real-world data
Source: Front Oncol. 2023 Mar 21;13:1043683. doi: 10.3389/fonc.2023.1043683 (PMC10072205; doi:10.3389/fonc.2023.1043683)
Supplement: Supplementary file 1 [file Table_1.docx]

|  | CM-067 | CHUV-RWD |
| --- | --- | --- |
| Five-year OS rates : |  |  |
| CTLA4 | 26% | 30% |
| PD1 | 44% | 38% |
| CTLA4+PD1 | 52% | 52% |
| Five-year OS Hazard ratio (95%CI and log-rank p-value): |  |  |
| PD1 vs CTLA4 | 0.63 (0.52 to 0.76; P<0.01) | 0.78 (0.49 to 1.26; P=0.31) |
| CTLA4+PD1 vs CTLA4 | 0.52 (0.42 to 0.64;P<0.01) | 0.55 (0.35 to 0.86; P<0.01) |
| CTLA4+PD1 vs PD1 | 0.83 (0.67 to 1.03;*) | 0.72 (0.45 to 1.13; P=0.15) |
| Five-year PFS rates : |  |  |
| CTLA4 | 8% | 11% |
| PD1 | 29% | 13% |
| CTLA4+PD1 | 36% | 29% |
| Five-year PFS Hazard ratio (95%CI and log-rank p-value): |  |  |
| PD1 vs CTLA4 | 0.53 (0.44 to 0.64; P<0.01) | 0.64 (0.42 to 0.98; P=0.04) |
| CTLA4+PD1 vs CTLA4 | 0.42 (0.35 to 0.51;P<0.01) | 0.52 (0.35 to 0.77; P<0.01) |
| CTLA4+PD1 vs PD1 | 0.79 (0.64 to 0.96;*) | 0.82 (0.57 to 1.19; P=0.29) |
| objective response rates : |  |  |
| CTLA4 | 19% | 22% |
| PD1 | 45% | 42% |
| CTLA4+PD1 | 58% | 53% |

Supplementary Table 1. Comparison of clinical outcomes between CM-067 and CHUV-RWD. * P-Values were not published.
