# Supplementary material for: Interactive process mining of cancer treatment sequences with melanoma real-world data
Source: Front Oncol. 2023 Mar 21;13:1043683. doi: 10.3389/fonc.2023.1043683 (PMC10072205; doi:10.3389/fonc.2023.1043683)
Supplement: Supplementary file 2 [file Table_2.docx]

|  | DREAMseq | CHUV-RWD |
| --- | --- | --- |
| Two-year OS rates (95% CI): |  |  |
| ICI→TT | 72% (63 to 79) | 63% (51 to 76) |
| TT→ICI | 52% (42 to 60) | 39% (22 to 56) |
| Two-year OS difference, Z-score: |  |  |
| ICI→TT vs TT→ICI | 20%, 3.16 | 24%, 2.23 |
| Two-year PFS rates (95% CI): |  |  |
| ICI→TT | 42% (31 to 52) | 28% (16 to 39) |
| TT→ICI | 19% (12 to 28) | 5% (0 to 13) |
| Two-year PFS difference, Z-score: |  |  |
| ICI→TT vs TT→ICI | 23%, * | 23%, 3.30 |

Supplementary Table 2. Comparison of clinical outcomes between DREAMSeq and CHUV-RWD. * Z-score not published.
